# Treatment of Unspecific Back Pain in Children and Adolescents: Results of an Evidence-Based Interdisciplinary Guideline

**DOI:** 10.3390/children9030417

**Published:** 2022-03-15

**Authors:** Michael Frosch, Stina Leinwather, Stefan Bielack, Susanne Blödt, Uta Dirksen, Michael Dobe, Florian Geiger, Renate Häfner, Lea Höfel, Bettina Hübner-Möhler, Thekla von Kalle, Burkhard Lawrenz, Andreas Leutner, Frauke Mecher, Kiril Mladenov, Heike Norda, Lorin Stahlschmidt, Marc Steinborn, Ralf Stücker, Ralf Trauzeddel, Regina Trollmann, Julia Wager, Boris Zernikow

**Affiliations:** 1 German Paediatric Pain Centre, Children’s and Adolescents’ Hospital, 45711 Datteln, Germany; s.leinwather@kinderklinik-datteln.de (S.L.); m.dobe@kinderklinik-datteln.de (M.D.); b.huebner-moehler@deutsches-kinderschmerzzentrum.de (B.H.-M.); l.stahlschmidt@deutsches-kinderschmerzzentrum.de (L.S.); j.wager@deutsches-kinderschmerzzentrum.de (J.W.); b.zernikow@kinderklinik-datteln.de (B.Z.); 2 Department of Children’s Pain Therapy and Paediatric Palliative Care, Faculty of Health, School of Medicine, Witten/Herdecke University, 58448 Witten, Germany; 3 Klinikum Stuttgart, Olgahospital, Stuttgart Cancer Center, Center for Pediatric, Adolescent and Women’s Medicine, Pediatrics 5 (Oncology, Hematology, Immunology), 70174 Stuttgart, Germany; s.bielack@klinikum-stuttgart.de; 4 Arbeitsgemeinschaft der Wissenschaftlichen Medizinischen Fachgesellschaften-Institut für Medizinisches Wissensmanagement (AWMF-IMWI), Philipps-Universität Marburg, 35043 Marburg, Germany; bloedt@awmf.org; 5 Pediatrics III, West German Cancer Center, University Hospital Essen, 45147 Essen, Germany; uta.dirksen@uk-essen.de; 6 Spine Center, Hessing Foundation, 86199 Augsburg, Germany; florian.geiger@hessing-stiftung.de; 7 German Center for Pediatric and Adolescent Rheumatology and Center for Pain Therapy for Young People, 82467 Garmisch-Partenkirchen, Germany; haefner.renate@rheuma-kinderklinik.de (R.H.); hoefel.lea@rheuma-kinderklinik.de (L.H.); 8 Department of Pediatric Radiology, Olgahospital, Klinikum Stuttgart, 70174 Stuttgart, Germany; t.vonkalle@klinikum-stuttgart.de; 9 Private Practice for Paediatrics and Adolescent Medicine, 59821 Arnsberg, Germany; blawrenz@t-online.de; 10 Department of Pediatric Surgery, Medical Center Dortmund, 44137 Dortmund, Germany; andreas.leutner@klinikumdo.de; 11 Physio Deutschland, German Federal Association for Physiotherapy, 50679 Cologne, Germany; info@physio-deutschland.de; 12 Department of Pediatric Orthopaedic Surgery, Children’s Hospital Hamburg-Altona, University Medical Center Hamburg-Eppendorf, 22763 Hamburg, Germany; kiril.mladenov@kinderkrankenhaus.net (K.M.); ralf.stuecker@kinderkrankenhaus.net (R.S.); 13 SchmerzLOS e.V. (Patient Advocacy Group), 23556 Lübeck, Germany; norda@schmerzlos-ev.de; 14 Department of Diagnostic and Interventional and Pediatric Radiology, München Klinik Schwabing, 80804 Munich, Germany; marc.steinborn@klinikum-muenchen.de; 15 Department of Pediatrics, Pediatric and Adolescent Rheumatology, Helios Klinik Berlin-Buch, 13125 Berlin, Germany; ralf.trauzeddel@helios-gesundheit.de; 16 Department of Pediatrics, Division of Neuropaediatrics, Friedrich-Alexander-Universität Erlangen-Nürnberg, 91054 Erlangen, Germany; regina.trollmann@uk-erlangen.de

**Keywords:** guideline, back pain, treatment, prevention, children, adolescents, evidence-based

## Abstract

Using a structured approach and expert consensus, we developed an evidence-based guideline on the treatment and prevention of non-specific back pain in children and adolescents. A comprehensive and systematic literature search identified relevant guidelines and studies. Based on the findings of this literature search, recommendations on treatment and prevention were formulated and voted on by experts in a structured consensus-building process. Physical therapy (particularly physical activity) and psychotherapy (particularly cognitive behavioral therapy) are recommended for treating pediatric non-specific back pain. Intensive interdisciplinary treatment programs should be provided for chronic and severe pain. Drug therapy should not be applied in children and adolescents. Further research on non-specific back pain in childhood and adolescence is strongly needed to reduce the imbalance between the high burden of non-specific back pain in childhood and adolescence and the low research activity in this field.

## 1. Introduction

As the leading cause of years lived with disability since 1990, low back pain represents a global health problem [1,2]. Even in children and adolescents, pain in the back is a serious health concern with a monthly prevalence of about 20%, increasing with age up to 18 years, to a lifetime prevalence of approximately 40% [3]. A recent large international study further revealed an increasing trend in the prevalence of chronic back pain in adolescents over the last two decades [4]. Between 12% and 20% of affected children and adolescents visit a health care professional for their back pain [5,6,7,8] and approximately 30% take pain medication [5,9]. The burden on the health care system and society is further increased as back pain in adolescence is a risk factor for adult low back pain [10], which is responsible for a considerable amount of work absenteeism [11]. The individual burden in childhood and adolescence includes restrictions in school and leisure activities and poor well-being [6,9,12,13,14]. Effective evidence-based treatment and prevention is urgently needed for this global health problem.

Mostly, in adolescence, there is no specific underlying condition causing the complaint, i.e., the back pain is non-specific [15]. There are several diagnostic measures (e.g., X-ray, magnetic resonance imaging) and risk factors (e.g., age, sex, psychosocial factors) that help isolate a specific cause of the back pain or assess whether the back pain is non-specific (see Frosch et al. in this special issue for a diagnostic algorithm). In the case of non-specific back pain, there are a variety of treatment options, such as massages, manual therapy, acupuncture or medication [16]. While evidence-based guidelines exist for adults [16,17], to date, there is no comprehensive evidence-based guideline for the treatment of non-specific back pain for children and adolescents. Such a guideline would be beneficial for health professionals, parents and affected youth to select the best available treatment.

Therefore, we developed an evidence-based guideline for back pain specifically in children and adolescents with the participation of all relevant German expert associations. This guideline provides information about the causes of specific back pain, risk factors for non-specific back pain and a diagnostic algorithm to differentiate specific from non-specific back pain (see Frosch et al. in this special issue). Furthermore, it includes evidence-based recommendations for different types of treatment and preventing pediatric back pain. These treatment recommendations reported here address both the early course of non-specific back pain and the persistent and chronic course of the condition in childhood and adolescence.

## 2. Materials and Methods

The guideline was developed based on the regulations of the Arbeitsgemeinschaft der Wissenschaftlichen Medizinischen Fachgesellschaften (AWMF (Association of Scientific Medical Societies in Germany); [18]). This contained a constituting meeting, a systematic literature search, an interdisciplinary structured consensus building and an external review.

### 2.1. Constituting Meeting

The guideline-working group consisted of representatives of 14 professional societies, organizations and patient representatives. In a constituting meeting held in April 2018, these representatives determined relevant topics and questions, the methodical approach to the systematic search and the appraisal of the quality of evidence. Working groups were established for the different topics.

### 2.2. Systematic Literature Search

The systematic literature search comprised two parts. First, existing guidelines on back pain in children and adolescents were searched in the relevant guideline databases (AWMF, Ärztliches Zentrum für Qualität in der Medizin (ÄZQ (German Agency for Quality in Medicine)), Guidelines International Network (GIN), National Institute for Health and Care Excellence (NICE), Scottish Intercollegiate Guidelines Network (SIGN), National Guidelines Clearinghouse (NGC)) on 6 February 2018 and again on 13 January 2021 due to the long processing period. Search terms were variations of “back pain” and “children and adolescents”. Eight guidelines were identified (see Supplementary Table S1), none of which met the rigorous standard needed for this guideline. Thus, our recommendations do not consider existing guidelines.

Second, on 18 April 2018, a systematic literature search was conducted in Medline, Central and Scopus. All databases were searched since their inception. Due to the limited number of comprehensive systematic reviews, individual studies on back pain in children and adolescents were screened as well and assigned to the different topics of the guideline. Search terms were combinations of variations of “back pain” and “children and adolescents” (see Supplementary Table S2 for search strategy). Again, due to the long processing period, on 28 January 2021 the literature search of treatment studies was updated. This update included a search in Medline of the original search terms that was limited to randomized controlled trials (RCT) and systematic reviews. Original articles, systematic reviews and meta-analyses published in English or German were included if they investigated back pain in children and adolescents between the ages of 3 and 18 years. Regarding treatment, only studies on non-specific back pain were included. All titles and abstracts were screened for these inclusion criteria. For potentially relevant articles, or when inclusion/exclusion could not be determined from the title or abstract, full texts were assessed. All titles, abstracts and full texts were screened by one person. Approximately 20% of the records were additionally screened by the first author (M.F.). The quality of the included articles was assessed with the Oxford Centre for Evidence-Based Medicine 2011 Levels of Evidence (OCEBM2011; [19]). Only studies with evidence levels 1 or 2 were included for further analyses. For treatment studies, risk of bias was assessed with A Measurement Tool to Assess Systematic Reviews (AMSTAR 2; [20]; systematic reviews), the Cochrane Risk of Bias Tool ([21]; RCTs) or the four-item checklist by Ballard & Montgomery ([22]; systematic review of systematic reviews). For included studies, relevant data were extracted according to the regulations of the AWMF [18]. For treatment studies, this included information on study type, population, interventions, outcomes, results, as well as strengths and weaknesses of the study.

**Figure 1:**
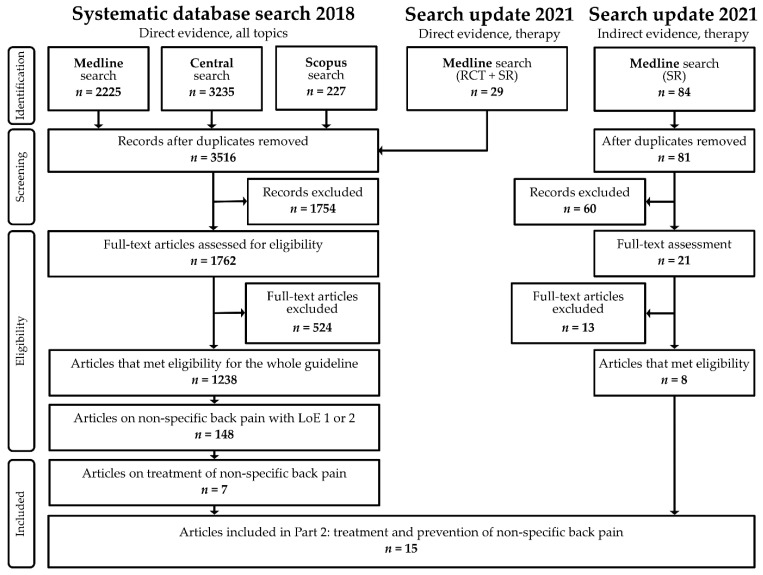
Flowchart. RCT = randomized controlled trial; SR = systematic review; LoE = level of evidence.

Some treatments had no associated articles. Therefore, the guideline-working group opted to conduct a search for indirect evidence on treatment of general chronic pain in children and adolescents. The working group explicitly decided against the use of indirect evidence from adult back pain treatment because of substantial differences in the treatment of adults versus children/adolescents. This search for indirect evidence was conducted on 28 January 2021 and included the original search terms for children and adolescents as well as “chronic pain”. Results of this additional search were limited to systematic reviews and processed as described above. Figure 1 displays the results of the systematic literature searches.

### 2.3. Consensus Building

Based on the results of the literature search, the respective working group formulated statements and recommendations for each topic. In March 2021, a Delphi round was completed to vote on the first draft of these statements and recommendations. Participants could approve a statement/recommendation or propose an amendment.

A final online consensus conference was held on 15 and 22 April 2021. Statements and recommendations that were not fully approved with 100% consensus in the Delphi round were discussed, reformulated if necessary, and again voted on. The whole guideline-working group participated in the Delphi round and the consensus conference. Each participating society or organization could only vote once, even if there were two representatives.

All members of the guideline-working group reported potential conflicts of interest. In cases where a conflict of interest was evident, the respective member abstained from voting on the relevant topic.

Based on the regulations of the AWMF [18], the strength of a recommendation could be classified as a “strong recommendation” (A), “recommendation” (B), or “open recommendation” (0). If evidence was missing, recommendations were based on expert consensus.

### 2.4. External Review

The final draft of the guideline was consulted and finally endorsed by the board of every participating association. Additionally, the draft was available for four weeks on the website of the AWMF for public consultation.

## 3. Results

Overall, 148 studies on non-specific back pain in childhood and adolescence were included for this guideline. Of these, only seven studies concerned treatment methods. Eight studies on general chronic pain in children and adolescents were added as indirect evidence. Thus, this guideline includes eight studies on non-drug therapies, four studies on drug therapy, two studies on interdisciplinary programs, two studies on prevention, and no studies on invasive therapy.

Specific recommendations for different treatment methods are presented below. Initially, the guideline-working group discussed general recommendations regarding treatment goals and the therapeutic process. As a result, expert consensus was 100% regarding the maintenance or restoration of the following treatment goals for non-specific back pain in children and adolescents:

- Normal everyday activities
- Physical activity and sports
- School attendance
- Social activities in leisure time

**Figure 2:**
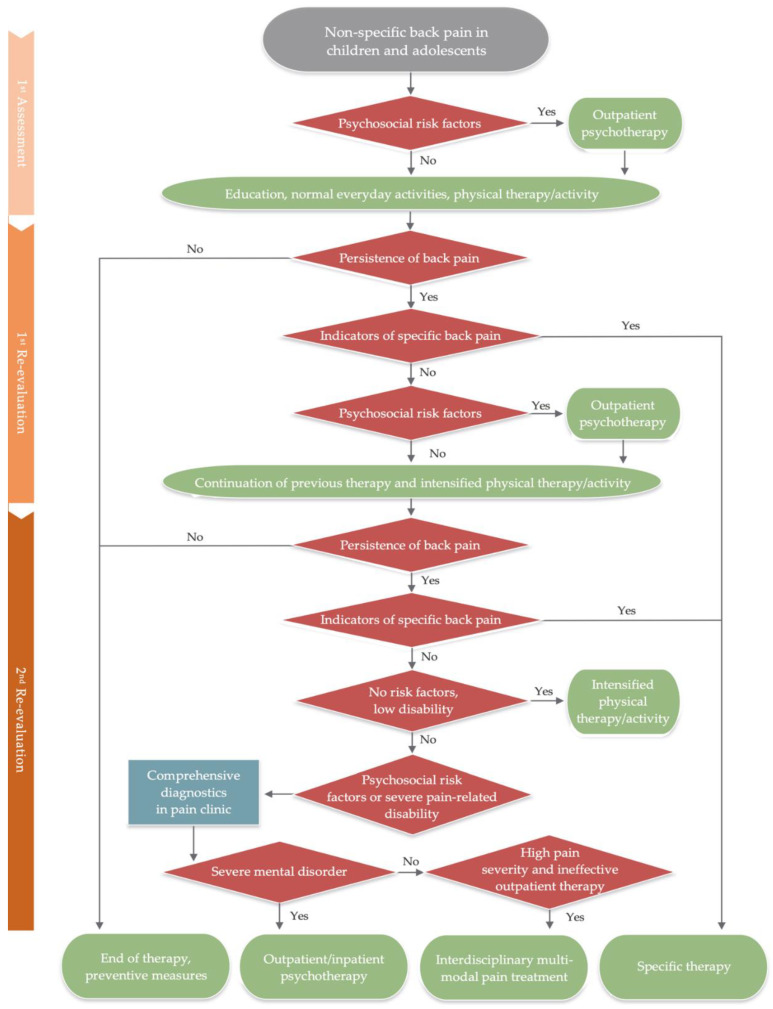
Therapeutic process after first assessment of non-specific back pain in children and adolescents. 1st re-evaluation 3–6 weeks after first assessment; 2nd re-evaluation 3 months after first assessment. Green box = treatment; blue box = diagnostic measures; red box = diagnostic decision.

Figure 2 describes the finalized therapeutic process.

### 3.1. Physical Therapy and Physical Activity

**Recommendation 1**: We recommend that active physical therapy be provided to children and adolescents with non-specific back pain. *(A*, *level of evidence 2, consensus 100%)*.

**Recommendation 2:** We recommend that in physical therapy, children and adolescents with non-specific back pain should be instructed to perform home exercise and physical activity. Adherence should be regularly monitored and adjusted by physical therapists. *(A*, *expert consensus 100%)*.

In a systematic review of systematic reviews, two high quality reviews confirmed a clinically meaningful positive effect of physical therapy in the treatment of non-specific back pain in children and adolescents [3]. Only limited statements or recommendations can be made about the exact physiotherapeutic method and duration of application. The duration of treatment in these studies ranges from four to 12 weeks, with an average of two applications per week. For many physical therapies, additional home exercise is an essential component.

**Recommendation 3:** Regarding manual therapy, no specific recommendation for non-specific back pain in childhood and adolescence can be provided due to inconsistent evidence. *(0, level of evidence 2, consensus 100%)*.

In a systematic review of systematic reviews, one systematic review with low methodological quality failed to demonstrate the effectiveness of manual therapy in the treatment of non-specific back pain in children and adolescents [3]. Yet, another systematic review of manual therapy concluded that manual therapy has a moderately positive impact on non-specific back pain in adolescence [23]; of the four studies included, however, only one RCT demonstrated that additional manual therapy reduced pain severity over the course of one year compared with the control group with only exercise therapy [23]. In another recent RCT of students with non-specific back pain, there were no differences between students who received instructions for exercises plus manual therapy in comparison to the control students given only exercise instructions [24]. Overall, due to contradictory results, currently, no recommendation for manual therapy can be made.

### 3.2. Psychotherapy

**Recommendation 4:** We endorse cognitive behavioral therapy (CBT) as the primary treatment for children and adolescents with recurrent or chronic non-specific back pain. *(B*, *level of evidence 2, consensus 100%)*.

No study examines the effectiveness of psychotherapy for non-specific back pain in children and adolescents. One Cochrane Review examined the effectiveness of cognitive and behavioral treatments on pain intensity and pain-related disability in children and adolescents with recurrent or chronic pain, including musculoskeletal pain [25]. Compared with active treatment, waiting list, or standard care, this review found a positive effect of cognitive and behavioral treatments on pain-related disability at follow-up (3 to 12 months after treatment) and on pain intensity post-treatment [25]. Additionally, a significant post-treatment reduction in anxiety symptoms but not in depressive symptoms was found [25]. Another meta-analysis found that psychological interventions reduced symptom load, disability and school absence in children with functional somatic symptoms [26]. However, only nine of the included 21 studies concerned chronic pain.

### 3.3. Drug Therapy

**Recommendation 5:** We advise against pharmacological treatment of recurrent or chronic non-specific back pain in children and adolescents. *(A*, *level of evidence 1–2, consensus 100%)*.

No studies have examined the effectiveness of drug therapy for non-specific back pain in children and adolescents. Additionally, for chronic non-cancer pain, the number of RCTs is limited. No studies have examined the harms and efficacy of acetaminophen (paracetamol) or opioids for this age group [27,28]. For nonsteroidal anti-inflammatory drugs (NSAIDs), one review identified seven controlled trials involving only patients with juvenile idiopathic arthritis [29]. An evidence-based assessment of risks and benefits of NSAIDs was not possible due to insufficient data [29]. Another systematic review of systematic reviews on co-analgesics for pain management in children and adolescents has identified a limited number of RCTs that include amitriptyline, serotonin antagonists and gabapentin [30]. In summary, there is no established evidence for the use of co-analgesics for recurrent or chronic pain in children and adolescents [30].

### 3.4. Invasive Therapy

**Recommendation 6:** We advise against invasive treatment for recurrent or chronic non-specific back pain in children and adolescents. *(A*, *expert consensus 100%)*.

The systematic literature search did not reveal any studies on invasive therapy for children and adolescents with non-specific back pain or general chronic pain. Thus, there is neither support for its efficacy nor a benefit-risk assessment in this patient population. To avoid treatment-induced adverse events or harm from invasive therapy, the guideline-working group does not recommend invasive therapy in children and adolescents with non-specific back pain.

### 3.5. Interdisciplinary Treatment Programs

**Recommendation 7:** We recommend that children and adolescents with recurrent and chronic non-specific back pain, severe pain-related disability, and ineffective unimodal treatment receive intensified interdisciplinary multimodal pain treatment. *(A*, *level of evidence 2, consensus 100%)*.

The systematic literature search found no studies examining the effectiveness of interdisciplinary treatment programs for non-specific back pain in children and adolescents. Two systematic reviews of chronic non-cancer pain together included nine RCTs [28,29]. The studies analyzed in these two reviews confirm that interdisciplinary multimodal pain treatment improves pain intensity, pain-related disability, symptoms of anxiety and depression and school attendance [31,32].

### 3.6. Prevention

**Recommendation 8:** To prevent back pain in children and adolescents, we recommend providing education and instructions for active exercise or encouraging regular physical activity and endurance sports. *(B*, *level of evidence 2, consensus 100%)*.

In a systematic review of systematic reviews, two high-quality reviews confirmed that preventative interventions involving education and postural advice can improve back care knowledge and may impact behavior [3]. Unfortunately, very few studies have examined the effectiveness of preventative measures on pain prevalence. Two high-quality reviews assessing pain prevalence suggest such effects are marginal at best [3]. However, a recent large RCT demonstrated that a school-based intervention including education and active exercise reduces the number of back pain episodes and the risk of new back pain [33]. A large population-based study further confirmed that moderate physical activity (specifically endurance sports) protects against non-specific back pain [34].

## 4. Discussion

This guideline aimed to provide evidence-based recommendations for the treatment and prevention of non-specific back pain in children and adolescents. Physical therapy (e.g., physical activity, home exercise) as well as psychotherapy—particularly CBT—is recommended for treatments of non-specific pediatric back pain. Intensive interdisciplinary treatment programs should be implemented for chronic non-specific back pain. Importantly, this guideline recommends against using medication and invasive procedures to treat non-specific pediatric back pain. Instead, education and physical activity are recommended to prevent back pain in children and adolescents.

This guideline’s systematic literature review revealed a lack of high-quality research on the treatment of non-specific back pain in children and adolescents. There are only a limited number of systematic reviews, many of which have considerable methodological problems [3]. The most comprehensive overview of systematic reviews is provided by Kamper et al. [3], who conclude that, while highly prevalent and burdensome in children and adolescents, non-specific back pain is underrepresented in the literature. Furthermore, there is an imbalance between the large number of (non-pharmacological) therapy options for non-specific pediatric back pain and the limited number of treatments for which effectiveness has been studied. High-quality research on available treatment options is needed to evaluate risks and benefits and to guide clinical practice. Guideline recommendations can only be as good as the available evidence used to develop them.

The lack of high-quality research on the prevention of non-specific back pain in children and adolescents is particularly alarming in light of its increasing prevalence over the last years [4]. Due to the global burden of back pain, research efforts should prioritize the prevention of back pain early in childhood and adolescence. Furthermore, future prevention studies need to focus on relevant outcomes. The studies included in this guideline mainly demonstrated that preventative measures can improve back care knowledge [3]. However, improving knowledge does not automatically improve behavior. Likewise, behavior changes may not directly reduce back pain episodes. Thus, future studies should particularly investigate the impact of preventative measures on back pain prevalence. A recent systematic review on school-based interventions confirmed that the combination of exercise and education—as recommended in this guideline—reduces back pain intensity, prevalence and frequency [35]. The importance of exercise is further supported by two other recent systematic reviews that identify sedentary behavior and being very low/high physically active as risk factors for low back pain in children and adolescents [36,37].

None of the treatment recommendations in this guideline are based on the best quality of evidence possible. Direct evidence for non-specific back pain in children and adolescents is only available for physical therapy, manual therapy and prevention. However, these studies only have an evidence level of two. Only the recommendations for drug therapy include studies with an evidence level of one. However, these studies provide only indirect evidence referring to general chronic pain. Similarly, only indirect evidence with an evidence level of two was available for important interventions, such as psychotherapy and interdisciplinary treatment programs. Therefore, more research is needed on these treatment domains, and results should be reported separately for different pain locations. This would facilitate the use of research results for guidelines on a specific type of pain and would greatly strengthen their evidence base.

While guidelines frequently serve to direct treatment planning in children and adolescents, they are often first developed for adults or based solely on adult research [14]. While understandable given the lack of evidence-based guidelines for this younger age group, our guideline reveals the need for separate assessments and recommendations for adults and children. There are two guidelines in Germany for non-specific low back pain for adults that recommend the temporary use of opioids or NSAIDs [16,38]. Likewise, non-selective NSAIDs and opioids are suggested for the treatment of low back pain in adults in a recent international guideline [17]. The present guideline for children and adolescents advises against the use of any drugs for non-specific back pain treatment. This recommendation was made, because either existing studies did not confirm drug efficacy or because studies were lacking that could reveal possible adverse events. Specific guidelines for children and adolescents are urgently needed to avoid medically induced harm. At the very least, sections for children and adolescents should be added to guidelines to point out differences in pediatric treatment.

A strength of this guideline is its high methodological quality. This includes the transparent handling of conflicts of interest, its structured approach to evidence collection, and its clear procedure according to approved regulations [18]. Still, recommendations are only as strong as the evidence guiding them. The evidence currently available is weak overall. Furthermore, AWMF recommends that patient representatives be included in the development of guidelines [18]. Despite great efforts to engage present or former patients, they declined the opportunity. Possible reasons for this unwillingness to participate could be the lack of a direct personal benefit or the expected significant time commitment. Additionally, active participation in the guideline could have counteracted successful pain therapy, namely diverting attention away from the pain. In the end, an adult patient representative participated in the working group.

This guideline’s comprehensive approach and inclusion of specific back pain in diagnostics resulted in a large number of studies that required extensive screening and processing. Future updates of this guideline will be less time-consuming as they will be able to focus on individual sections instead of all historical literature.

The present guideline summarizes treatment recommendations for non-specific back pain in children and adolescents. Although research for this age group is scarce, this guideline provides recommendations based on the best available evidence. Physical activity and CBT are recommended for pediatric back pain treatment, as well as interdisciplinary treatment programs for chronic courses. In contrast to adult treatment, children and adolescents should not receive medication. Future research on non-specific back pain in childhood and adolescence is strongly needed.

## Supplementary Materials

The following supporting information can be downloaded at: https://www.mdpi.com/article/10.3390/children9030417/s1, Table S1: Results of the systematic guideline search; Table S2: Medline search strategy.
