# Supplementary material for: Treatment of Unspecific Back Pain in Children and Adolescents: Results of an Evidence-Based Interdisciplinary Guideline
Source: Children (Basel). 2022 Mar 15;9(3):417. doi: 10.3390/children9030417 (PMC8947172; doi:10.3390/children9030417)
Supplement: Supplementary file 1 [file children-09-00417-s001.zip › children-1542655-supplementary.pdf]

children

Supplementary Table S1. Results of the systematic guideline search.

No.	Identified guideline	Available online	Reason for exclusion
1	ACR Appropriateness Criteria® back pain—child (2016)	sciencedirect.com/science/article/pii/S1546144017301461	Not evidence-based (recommendations not linked to references)
2	Definition, Pathophysiologie, Diagnostik und Therapie des Fibromyalgiesyndroms	awmf.org/leitlinien/detail/ll/145-004.html	Not non-specific back pain
3	European guidelines for prevention in low back pain - On behalf of the EU-COST B13 Working Group (2004)	https://www.ncbi.nlm.nih.gov/pmc/articles/PMC3454541/pdf/586_2006_Article_1070.pdf	Outdated, some documents not findable, evidence-base not verifiable
4	Langzeitanwendung von Opioiden bei chronischen nicht-tumorbedingten Schmerzen (LONTS) - S3	awmf.org/leitlinien/detail/ll/145-003.html	No evidence-based recommendations for children and adolescents
5	Management of Chronic Pain in Children and Young People (2018)	sign.ac.uk/media/1538/chronic_pain_in_childrenpdf.pdf	Not evidence-based (recommendations not linked to references)
6	Muskuloskelettale Schmerzen bei Kindern und Jugendlichen: Algorithmus zur differenzialdiagnostischen Abklärung eines onkologischen Leitsymptoms - S2K	awmf.org/leitlinien/detail/ll/027-073.html	Not evidence-based
7	Pain in the back – Avoiding back pain in children and teenagers	https://www.erwcpt.eu/file/295	No guideline
8	Rückenschmerz (nicht traumatisch) bei Kindern - Bildgebende Diagnostik - S1	awmf.org/leitlinien/detail/ll/064-012.html	Not evidence-based

Supplementary Table S2. Medline search strategy.

No.	Search term	Search fields
1	Child	Title/Abstract
2	Child*	Title/Abstract
3	Adolescen*	Title/Abstract
4	Teen	Title/Abstract
5	Teen*	Title/Abstract
6	Youth	Title/Abstract
7	Youth*	Title/Abstract
8	Pediatri*	Title/Abstract
9	Paediatri*	Title/Abstract
10	1 OR 2 OR 3 OR 4 OR 5 OR 6 OR 7 OR 8 OR 9	
11	Back pain	Title/Abstract
12	Back pains	Title/Abstract
13	Back ache	Title/Abstract
14	Back aches	Title/Abstract
15	Backache	Title/Abstract
16	backaches	Title/Abstract
17	11 OR 12 OR 13 OR 14 OR 15 OR 16	
18	10 AND 17	

* wildcard to search for all possible endings.
